# The Rising Role of Omics and Meta-Omics in Table Olive Research

**DOI:** 10.3390/foods12203783

**Published:** 2023-10-15

**Authors:** Anastasios Tsoungos, Violeta Pemaj, Aleksandra Slavko, John Kapolos, Marina Papadelli, Konstantinos Papadimitriou

**Affiliations:** 1 Department of Food Science and Technology, University of the Peloponnese, 24100 Kalamata, Greece; a.tsouggos@go.uop.gr (A.T.); v.pemaj@go.uop.gr (V.P.); a.slavko@go.uop.gr (A.S.); i.kapolos@uop.gr (J.K.); m.papadelli@uop.gr (M.P.); 2 Laboratory of Food Quality Control and Hygiene, Department of Food Science and Human Nutrition, Agricultural University of Athens, Iera Odos 75, 11855 Athens, Greece

**Keywords:** fermentation, microbiome, genomics, transcriptomics, proteomics, metagenomics, metabolomics, volatiles, metabolites

## Abstract

Table olives are often the result of fermentation, a process where microorganisms transform raw materials into the final product. The microbial community can significantly impact the organoleptic characteristics and safety of table olives, and it is influenced by various factors, including the processing methods. Traditional culture-dependent techniques capture only a fraction of table olives’ intricate microbiota, prompting a shift toward culture-independent methods to address this knowledge gap. This review explores recent advances in table olive research through omics and meta-omics approaches. Genomic analysis of microorganisms isolated from table olives has revealed multiple genes linked to technological and probiotic attributes. An increasing number of studies concern metagenomics and metabolomics analyses of table olives. The former offers comprehensive insights into microbial diversity and function, while the latter identifies aroma and flavor determinants. Although proteomics and transcriptomics studies remain limited in the field, they have the potential to reveal deeper layers of table olives’ microbiome composition and functionality. Despite the challenges associated with implementing multi-omics approaches, such as the reliance on advanced bioinformatics tools and computational resources, they hold the promise of groundbreaking advances in table olive processing technology.

## 1. Introduction

Fermented foods are products deriving from bioconversion, during which microorganisms transform the components of the raw material, modifying their flavor, texture, and nutritional properties into the final product [1,2,3,4]. It was realized early on that the fermentation process is a simple and direct way of preserving food per se [3,5]. Fermented foods have been part of the human diet for thousands of years, with evidence of their consumption dating back to ancient civilizations [4,6,7]. In recent years, there has been a renewed interest in fermented foods, given the fact that we can study them in unprecedented depth due to many different scientific advances over the past decade (please see below) [8,9]. In addition, their potential health benefits have gained much attention since they are being evidenced systematically in a wide range of physiological situations and diseases [8,9,10]. Nowadays, there are a multitude of studies suggesting that fermented foods may help to promote gut health, boost the immune system, improve digestion, prevent obesity, lower the risk of cardiovascular diseases and diabetes or even affect emotions and mental awareness via the gut–brain axis [2,3,11]. Nevertheless, consumption of fermented foods may also involve health risks. Considering that fermentation is sometimes an uncontrolled process, pathogenic microorganisms, such as *Clostridium botulinum* and *Staphylococcus aureus*, might contaminate the food and expose consumers to serious infections [12].

Table olives are one of the most popular plant-based fermented foods in the Mediterranean, with an increasing trend of consumption worldwide [13]. The global production corresponds to approximately three million tons per year, with Spain, Greece, Italy and Portugal being the top producers in Europe. Some of the most notable varieties of table olives are Manzanilla, Sevillana, Hojiblanca, Ascolana Tenera, Picholine, Cerignola, Kalamata, Halkidiki, Conservolea, Gemlik and Ayvalik [14].

**Figure 1:**
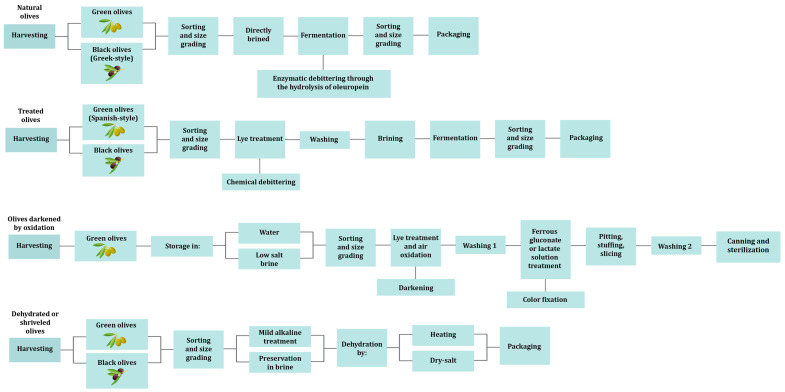
Table olive processing methods.

Olive fruits are primarily composed of water (60–75%), lipids (10–25%), low concentration of sugars (2–5%), and phenolic compounds (1–3%), that are mainly consisting of oleuropein [15]. Oleuropein is responsible for the bitter taste and all methods to produce edible olives involve at least one step of debittering [16,17]. Based on the processing steps, table olives are divided into four different trade preparations according to International Olive Council (IOC), i.e., the treated olives, the natural olives, the olives darkened by oxidation and the dehydrated or shriveled olives (Figure 1) [18]. Treated olives are initially subjected to alkaline treatment for debittering and then a brine solution is added for partial or complete fermentation [14,15,18,19,20]. Primarily, olives at the green stage of maturation are used and this preparation is called Spanish-style. Natural olives are directly brined and the debittering takes place during fermentation through the enzymatic hydrolysis of oleuropein [14,15,18,20]. Usually, olives at the black stage are used and this preparation is known as Greek-style. The olives darkened by oxidation, also called California-style or black ripe olives undergo lye treatment after harvesting and then the olives are placed into a container with water (or low-salt brine) and are continuously aerated [7,14,18]. Through this process the olives take a uniform black color due to oxidation. This type of table olives has to be heat treated to be considered safe for consumption [21]. Lastly, in the dehydrated or shriveled table olives, the olives may undergo a mild alkaline treatment and then are dehydrated in dry salt (dry-salted) and/or by heat treatment [18,20]. In these conditions, fermentation is very difficult to occur leading to dry salting being identified as a “curing” method [22,23]. 

**Figure 2:**
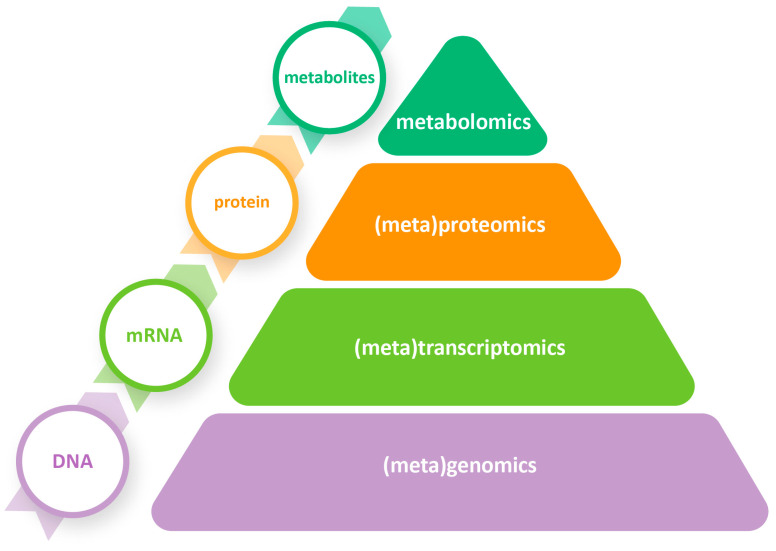
Omics and meta-omics approaches that can be applied to study table olives at different molecular levels.

Fermentation is a complex biochemical procedure involving multiple metabolic pathways and interactions between different microorganisms [5]. Classic culture-dependent techniques can only capture a small fraction of the complicated microbiota of fermented foods, and they can only provide limited information about the specific role of each microorganism [19]. Recent culture-independent methods, such as omics technologies, offer an in-depth understanding of the different mechanisms associated with the fermentation process [24]. Omics is a field of study that includes, among others, genomics, transcriptomics, proteomics, and metabolomics, with all their meta-omics derivatives, such as metagenomics, metatranscriptomics, metaproteomics, and meta-metabolomics (metabolomics from now on) (Figure 2).

Through omics, researchers can analyze the genomic, transcriptomic, proteomic, and metabolic profiles of pure microorganisms or entire microbial ecosystems, enabling a deeper understanding of their functional capabilities and interactions [24]. Single-omics approaches can provide more information about the fermentation process in comparison with classic approaches, including the dynamics and the functional properties of microbial consortia. Nevertheless, multi-omics approaches enable analysis at multiple levels, facilitating the exploration of fermentation at increasing levels of complexity via each separate omics approach employed [25]. Using these strategies, snapshots of food fermentations with unparallel detail have been achieved that hold the promise of revealing the entire mechanisms which underpin the process, not only at the laboratory scale but also in an industrial setting. 

In this review, we provide a comprehensive analysis of recent omics and meta-omics studies conducted on table olives. These cutting-edge applications are currently revolutionizing our understanding of microbial dynamics and their functional properties, which shape the characteristics and qualities of table olives. Delving into genomics, proteomics, transcriptomics, metabolomics, and metagenomics, our review offers an extensive view of the most recent findings and their implications in the field.

## 2. A Primer on the Fermentation Ecosystem of Table Olives

The study of table olive fermentation has been the focus of an important amount of literature prior to the advent of throughput omics, including a number of review articles. In most cases, it is a spontaneous process triggered by autochthonous microorganisms from the olives’ ecosystem, mostly lactic acid bacteria (LAB) and yeasts [26,27]. The role of LAB is to consume sugars and other nutrients of the olives and to produce organic acids as byproducts, lowering the pH of the brine and making it more acidic in order to create an environment that is unfavorable to spoilage and harmful bacteria [28]. In contrast to earlier reports, yeasts are now considered crucial for fermentation, as they can positively influence the organoleptic characteristics of olives and also exhibit a synergistic effect by helping the growth of LAB [20,29]. In some cases, yeasts can even become the prevailing population due to the presence of phenolic compounds, the high NaCl concentration (>8%) and the low pH values achieved during fermentation, leading to a final product with a lighter taste and shorter shelf life [15,30,31,32,33,34].

In general, the microbiota of table olives usually includes members of the family *Lactobacillaceae*, with the main representatives being the genera *Lactiplantibacillus*, *Lacticaseibacillus*, *Pediococcus*, *Leuconostoc* and *Enterococcus* [20,27], while among yeasts, *Candida*, *Pichia*, *Saccharomyces*, *Debaryomyces*, and *Wickerhamomyces* are the most common genera detected [26,29,30]. Molds are present mostly in natural black fermented olives, with *Penicillium* spp., *Aureobasidium* spp., *Aspergillus* spp. and *Geotrichum* spp. being the most frequently identified genera [26,35,36]. Molds in table olives can cause spoilage and affect the quality of the olives. However, molds have been suggested to contribute to the formation of the characteristic flavors and aromas of the final product [36]. Spoilage microorganisms, such as members of the family *Enterobacteriaceae* and the genera *Pseudomonas*, *Vibrio*, and *Clostridium*, can be found in olives in the early stages of fermentation, although the reducing pH combined with the presence of NaCl and the anaerobic conditions (at least for the aerobic bacteria) of a normal fermentation inhibit any further growth [26,37].

As was already mentioned, table olive fermentation can be a spontaneous process that is difficult to control because of the unpredictable nature of the microorganisms that are involved. Raw olives are not thermally treated, so the inhibition of spoilage microorganisms is based exclusively on the addition of salt, the development of anaerobic conditions and the presence of the fermenting microorganisms that will limit their growth [38]. The use of starter cultures is gaining a lot of interest, as it has the potential to control the fermentation process and result in a standardized product [39,40]. Starter cultures have been used mainly in relation to Spanish-style olives to prevent the development of spoilage microorganisms and/or to accelerate the acidification process [40,41]. Recently, there has been a growing trend toward replacing NaCl in table olive fermentation with other salts [38]. One reason for this is to reduce the sodium content of table olives, as a high sodium intake has been linked to several health issues, such as high blood pressure. Alternative salts such as KCl, CaCl_2_, and ZnCl_2_ have been tested; however, earlier studies reported that using alternative salts resulted in the altering of the table olives’ microbiota in comparison with the control samples treated with NaCl, which could lead to a negative impact on the organoleptic characteristics of the olives [42,43,44,45,46].

## 3. Genomics

Genomics is the study of an organism’s entire genome, focusing on the functionality, structure and interactions of the genes included [47]. Several genome-sequencing studies have been conducted on LAB isolated from table olives, aiming to uncover specific genes that are responsible for the potential probiotic properties or genes that could influence the process of fermentation. Perpetuini et al. [48] performed a genetic screening to identify the essential genes for growth in olives brines in the genome of *Lp. pentosus* C11. The findings clearly indicated that the *enoA1*, *gpi*, and *obaC* genes, encoding an enolase, a glucose-6-phosphate isomerase, and a putative fatty-acid-binding protein, respectively, were critical for growth in this type of environment. Abriouel et al. [49,50] reported the complete genome sequence of the potential probiotic *Lp. pentosus* MP-10 isolated from Aloreña green table olives. Subsequently, the *Lp. pentosus* MP-10 genome was analyzed to unravel the molecular mechanisms associated with safety aspects and the probiotic properties of this strain [51,52]. MP-10 was found to lack any antibiotic resistance genes, while a (clustered regularly interspaced short palindromic re-peats) CRISPR/cas system was identified which is an immune system against foreign genetic elements, such as viruses, transposable elements and conjugative plasmids [51]. Several genes involved in carbohydrate metabolism were identified, which could be crucial for the strain’s survival in the human gastrointestinal tract [52]. Furthermore, genes encoding for mucus-binding and moonlighting proteins were found, possibly being involved in their adhesion to epithelial cells and/or the extracellular matrix. In silico analysis of *Lp. pentosus* MP-10 plasmids (pLPE-1 to pLPE-5) demonstrated their significant contribution to the probiotic properties of the strain [53]. Plasmid-borne genes were found to play an important role in mucin adhesion, carbohydrate metabolism, metal tolerance and the bioremoval of several metals, a property that could be used to reduce toxic metal levels in the gut and food products or even in environmental bioremediation. In another study, genomic analysis of *Lp. pentosus* CF2-10N demonstrated the probiotic properties of this strain via the presence of genes encoding for proteins involved in adhesion, exopolysaccharide biosynthesis, bile salt resistance, tolerance to highly acidic pH, immunomodulation, and vitamin production [54]. Calero-Delgado et al. [55,56] presented the genome sequences of 11 *Lp. pentosus* strains (IG2 to IG12) isolated from table olive brines or biofilms on the skins of olives. Several genes encoding for proteins involved in bacteriocin and EPS production, as well as mucin-binding proteins, were found. Whole-genome sequencing (WGS) of *Lp. pentosus* LPG1 isolated from table olive biofilms confirmed the multifunctional features of this strain, as various genes involved in acid stress resistance, bile salt tolerance, carbohydrate metabolism and adhesion were found [57]. Pan-genome analysis of the LPG1 strain revealed that IG8, IG9, IG11, and IG12 were the more closely related strains, all of them isolated from table olive biofilms. Moreover, LPG1 and the IG8, IG9, IG11, and IG12 strains share the same genomic island (GI) that encodes for L-arabinofuranosidase and beta-galactosidase, suggesting a probable common ancestor among them. The screening of 77 different *Lactiplantibacillus* strains for technological properties by Zotta et al. [58] distinguished *Lp. pentosus* O17 as the strain that exhibited the highest potential, and therefore, the strain was subjected to WGS. All the key genes responsible for phenolic compound degradation and metabolism were found in the genome of the O17 strain, with the exception of carboxylesterase and gallate decarboxylase subunits B and D. Another comparative genomics analysis was performed by Garcia-Gonzalez et al. [59] between three *Lp. plantarum* strains isolated from the human GIT (IMC513), cheese (LT52) and table olives (C904). The genomic analysis of *Lp. plantarum* C904 revealed the presence of genes related to bacteriocin and EPS production, mucus-binding ability, stress tolerance and bile salt metabolism.

While genomic studies in the field of table olives usually focus on the analysis of LAB strains, there have been descriptions of other relevant bacterial or fungal genomes to a lesser extent as well. Camiolo et al. [60] conducted whole-genome sequencing (WGS) on eight *C. boidinii* strains, which were isolated from various environments, including four strains (UNISS-Cb18, UNISS-Cb60, TOMC-Y13, TOMC-Y47) that were specifically isolated from table olives. Several genes were found to be primarily associated with translation, transcription, signal transduction mechanisms, carbohydrate and amino acid metabolism and defense mechanisms. A novel *Aspergillus* species was isolated by Crognale et al. [61] from olive brine wastes. After phylogenetic and morphological analysis, the novel species, which was named *Aspergillus olivimuriae*, underwent WGS and a comparative analysis of the whole-genome orthologous genes was performed with *A. flavus*, *A. terreus* and *A. fumigatus*, showing 5929 shared orthologous genes among all the compared species. Furthermore, Pontes et al. [62] employed a comparative genomic and evolutionary analysis to confirm that *S. cerevisiae* populations from processed olives resulted from a hybridization between *S. cerevisiae* and *S. paradoxus*, and they also demonstrated the adaptation of these strains to olive brines, in contrast to wine strains, referring to this kind of adaptation as quasi-domestication.

## 4. Metagenomics

Metagenomics is the direct genetic analysis of the total microbial genomic DNA within a sample, without the need for individual isolation and cultivation of microorganisms [63]. It is divided into two major approaches. The first approach is targeted metagenomics (also called metataxonomics), which relies on the amplification of a specific gene (e.g., 16S rDNA, 18S rDNA, etc.) or a genomic region (e.g., the yeast internal transcribed spacer, ITS) and the subsequent sequencing of the amplicons. The second approach is untargeted (shotgun) metagenomics, which involves the sequencing of the entire genetic material in a sample without any prior knowledge of its content [64]. The metagenomics approach was initially applied in the field of table olives by Cocolin et al. [65], who investigated the effect of NaOH treatment on the bacterial ecology of Nocellara Etnea table olives, and several other studies have been performed since then. The metagenomics studies on table olives are summarized in Table 1.

### 4.1. Spanish-Style Olives

One of the first applications of a metagenomics approach on Spanish-style table olives was the study by de Castro et al. [66], investigating the bacterial and fungal populations of spoiled Gordal and Manzanilla table olives. The results showed the dominance of some unexpected bacterial taxa, such as *Cardiobacteriaceae* and *Ruminococcus*, while the most abundant fungal population turned out to be *P. membranifaciens*, which is considered common in table olives. In a similar experiment, Arroyo-Lopez et al. [67] focused on the microbiota of Manzanilla table olives affected by butyric, sulfidic, or putrid spoilage. The olives affected by butyric spoilage exhibited a distinctive abundance of *Enterococcus* that might have caused the defect. In contrast, the sulfidic samples showed an abundance of *Alkalibacterium* and *Marinilactibacillus* instead of *Lactiplantibacillus*, which was the prevailing genus in the normal fermentations. Lastly, in putrid spoilage, although the dominant genus was *Lactiplantibacillus*, high percentages of other genera, such as *Marinilactibacillus*, *Alkalibacterium*, *Paraliobacillus*, *Enterococcus*, and *Halomonas*, were also identified. The microbial diversity of Spanish-style Halkidiki olives from two different regions of Greece was studied by Argyri et al. [35]. In the majority of the samples, the prevailing genus was found to be (former) *Lactobacillus*, while in certain samples, *Pediococcus* prevailed against (former) *Lactobacillus*. In the same study, the yeast microbiota presented a homogenous population and *Pichiaceae* dominated in all the samples from both regions. These results were confirmed by Tzamourani et al. [68] in another research on Spanish-style table olives of the Halkidiki cultivar stored in modified atmosphere pouches. Fungal diversity was determined through ITS amplicon sequencing, and once again, the *Pichiaceae* family dominated in all the samples, with *Pichia manshurica* and *P. membranifaciens* being the prevailing species at the beginning of storage. As was mentioned earlier, there is an ongoing trend in Spanish-style table olives to use starter cultures as a method to control fermentation. Benitez-Cabello et al. [69] tested four different strains as starter cultures during Spanish-style fermentation of Manzanilla olives, investigating their microbiota after 65 days through a metataxonomic analysis. The utilization of starter cultures prevented the emergence of harmful or spoilage microorganisms, resulted in effective acidification and fermentation processes and promoted the growth of various bacterial genera, like (former) *Lactobacillus*, *Marinilactibacillus*, *Alkalibacterium*, and *Halolactobacillus*, in all the applications.

### 4.2. Natural Olives

#### 4.2.1. Natural Green Olives

Natural green olives, such as the Aloreña de Málaga variety, have been gaining increasing demand lately in comparison with the Spanish-style olives that had dominated the industry over the years. Although LAB have been proved to be crucial for the table olive fermentation process, initial studies in directly brined Aloreña de Málaga olives failed to identify LAB in significant abundance [70,71]. Instead, *Pseudomonas*, *Modestobacter*, *Acetobacter*, and *Propionibacterium* were the main genera observed in both olive and brine samples, while *Celerinatantimonas* was the predominant genus at the end of the process [71]. These results were updated by Rodriguez-Gomez et al. [72] in a study on heat-shocked Aloreña de Málaga olives in which (former) *Lactobacillus* (83.67%) and *Pediococcus* (12.30%) were the most abundant genera found through 16S rDNA amplicon sequencing. Sicilian-style table olives are another type of natural green olive, being directly brined in approximately 8% NaCl. Randazzo et al. [73], focusing on Sicilian-style Nocellara Etnea table olives inoculated with starter cultures or not, investigated through 16S rRNA gene-based analysis the bacterial microbiota at the beginning and at the end of fermentation. The results highlighted significant differences between the inoculated and the uninoculated olives at the beginning of fermentation, with *Halomonas*, *Achromobacter*, *Marinobacter*, and *Flavobacteriaceae* dominating in the uninoculated olives, while in the inoculated ones, (former) *Lactobacillus* prevailed. During fermentation, the uninoculated samples exhibited a significant increase in *Lactobacillaceae*, reaching a similar profile as the inoculated samples at the end of the process. Recently, Ruiz-Barba et al. [74,75] conducted two amplicon-sequencing studies on natural green table olives of different cultivars. In the Gordal table olives, the dominant bacterial genera were *Lactiplantibacillus* and *Pediococcus*; however, in the Hojiblanca and Manzanilla (in both studies) samples, all the detected bacterial genera were gram-negative, such as *Halomonas*, *Marinobacter* and *Alidiomarina*. Interestingly, members of the *Enterobacteriaceae* family were significantly abundant, especially in the Hojiblanca samples, even though they were not detected using the culturable techniques [75]. Regarding yeasts, *Saccharomyces*, *Pichia* and *Candida* were the prevailing genera in the Manzanilla samples (in both studies), the Gordal samples were characterized by the dominance of *Candida* and in the Hojiblanca samples, Nakazawaea was the most abundant yeast genus found. We believe that these findings are interesting, although the prevalence of gram-negative bacteria may need further investigation.

#### 4.2.2. Natural Black Olives (Greek-Style)

Initial culture-based studies of Kalamata olives reported that LAB were not detected during fermentation or that they were only detected in the early stages [76,77]. Nevertheless, amplicon sequencing by Kazou et al. [78] reported that *Lactobacillaceae* was the dominant family identified, with (former) *Lactobacillus* and *Leuconostoc* being the genera found in greater abundance. The diversity of yeast and fungal microbiota of the samples was significantly lower, and the prevailing genera were *Pichia*, *Saccharomyces*, *Penicillium*, and *Ogataea*. Conservolea is another olive cultivar usually fermented via the traditional Greek-style method similar to Kalamata olives. Amplicon-sequencing analysis showed that LAB were the dominant population in this olive cultivar too [35,68]. The authors suggested that the results obtained could be attributed to the low salt level (6–8%) used in this type of fermentation, which allows LAB to prevail against the yeast population [35]. In particular, (former) *Lactobacillus*, *Pediococcus*, and *Leuconostoc* were the genera that showed the greatest abundance. As far as yeasts are concerned, once again the predominant family was *Pichiaceae*, with the exception of the same samples from Magnesia in which *Phaffomycetaceae*, and more specifically *W. anomalus*, prevailed. In certain instances, members of the Staphylococcus and/or Enterobacter genera were also detected in some Greek-style olive samples [40,78,79]. It is interesting that no enterobacteria were found during culture-based analysis, but only through amplicon sequencing, which may be attributed to the presence of dead cells [46]. This hypothesis seems plausible since gram-negative bacteria are usually present in the early stages of black table olive fermentation, although they are gradually inhibited by the low pH values of the brines, as already mentioned [46,80].

The fermentation of table olives may be dictated exclusively by the indigenous microbial ecosystem of the olives [27]. The mapping of the microbial diversity in various olive varieties from different regions can enhance our understanding of table olive fermentation and its association with the olive variety and origin, which could also assist in the promotion of new table olive varieties as Protected Designation of Origin (PDO) or Protected Geographical Indication (PGI) products [79]. In the case of Greek-style table olives, both Argyri et al. [35] and Kazou et al. [78] managed to obtain promising results in discriminating table olives by variety and region of origin, increasing the interest in further research in the field. However, Kamilari et al. [79], while evaluating the microbial diversity among three table olive cultivars from different regions of Cyprus, failed to discriminate between them based on their bacterial diversity. Nevertheless, in the same study, fungal-based diversity seemed to be an effective tool to distinguish between table olive varieties.

Storage and packaging are also important parts of the table olive industry, as it has been shown that they affect the organoleptic characteristics and the microbial communities of the product. Modified atmosphere packaging (MAP) is a popular technique nowadays used to preserve the quality and shelf life of products; however, there have not been enough studies yet on its effect on the microbial population of the product [81]. Michailidou et al. [81] used 16S rRNA and 18S rRNA gene sequencing to monitor the gradual changes in the microbiome of table olives that were packaged under a modified atmosphere. The results were in agreement with previous studies on table olive microbiomes, as (former) *Lactobacillus* and *Pediococcus* for bacteria and *Pichia* for yeasts were the dominant genera identified. While during the first stages of storage the dominant bacterial species were *Pediococcus ethanolidurans* and *Lp. plantarum*, they were gradually displaced by *Lentilactobacillus parafarraginis* and *Lentilactobacillus buchneri* during the following stages. A similar effect of MAP was observed on the fungi population too, since *Brettanomyces* gradually replaced *Pichia* as the prevailing genus during the storage time.

### 4.3. California-Style Olives

California-style table olives darkened by oxidation are considered safe as they undergo sterilization during processing [18,82]. Prior to Medina et al. [82], there was limited knowledge regarding the microbial ecology of California-style table olives. Through 16S rRNA and ITS sequencing, it was shown that the bacterial and fungal populations change significantly through the processing steps of Manzanilla and Hojiblanca olives. More specifically, during preservation, (former) *Lactobacillus* dominated in the Manzanilla samples, while *Acetobacter* was the predominant genus in the Hojiblanca samples. During the first washing stage, *Acetobacter* and (former) *Lactobacillus* continued to be the predominant bacterial population throughout the samples. The addition of ferrous gluconate solution led to the replacement of (former) *Lactobacillus* by *Oenococcus* as the dominant LAB in the samples. Finally, sequencing identified over 20 bacterial genera in samples from the second washing step, which exhibited the greatest microbial diversity among all the processing stages. Regarding yeasts, the differences between the processing stages were less significant, with *Kregervanrija fluxuum* and *P. membranifaciens* dominating at the beginning and then members of the family *Dipodascaceae* taking over as the most abundant population found.

### 4.4. Dry-Salted Olives

Dry-salted olives are usually produced in Greece, Algeria, Turkey, Morocco and other Mediterranean countries [23]. The presence of LAB in dry-salted olives is not usually expected because of the low water activity and the high salt content of the product. Knowledge about the microbiota of dry-salted olives is relatively limited; however, Gounari et al. [22] investigated the yeast diversity of dry-salted naturally black olives (cv. Throuba Thassos) via amplicon target sequencing (ATS). The results revealed high yeast diversity, with the dominant species being *Candida etchellsii*, *Candida versatilis*, *P. membranifaciens*, *Candida apicola*, *Pichia triangularis* and *W. anomalu.*

**Table 1:** Metagenomics studies on table olives.

Processing Method	Variety	Genera	Reference
Spanish-style and natural green	Nocellara Etnea	*Chromohalobacter*, *Halomonas*, (former) *Lactobacillus*, *Chromohalobacter*, *Marinilactibacillus*	Cocolin et al. [65]
Spanish-style	Manzanilla and Gordal	*Suttonella*, *Dekkera*, *Ruminococcus*, *Pichia*, *Candida*	de Castro et al. [66]
Spanish-style	Manzanilla	*Lactiplantibacillus*, *Vibrio*, *Alkalibacterium*, *Marinilactibacillus*, *Halolactibacillus*, *Enterococcus*	Arroyo-Lopez et al. [67]
Spanish-style and Greek-style	Conservolea and Halkidiki	(former) *Lactobacillus*, *Pediococcus*, *Pichia*, *Wickerhamomyces*, *Brettanomyces*, *Aureobasidium*, *Schwanniomyces*	Argyri et al. [35]
Spanish-style	Conservolea and Halkidiki	*Pichia*, *Brettanomyces*, *Saccharomyces*, *Candida*, *Quambalaria*, *Aureobasidium*, *Rhodosporidiobolus*, *Cladosporium*	Tzamourani et al. [68]
Spanish-style	Manzanilla	(former) *Lactobacillus*, *Marinilactibacillus*, *Alkalibacterium*, *Halolactobacillus*	Benitez-Cabello et al. [69]
Greek-style	Kalamata	(former) *Lactobacillus*, *Leuconostoc*, *Pichia*, *Saccharomyces*, *Penicillium*	Kazou et al. [78]
Greek-style and natural green	Cypriot, Kalamata, Picual	*Lactobacillus*, *Streptococcus*, *Lactococcus*, *Lactiplantibacillus*, *Aspergillus*, *Candida*, *Botryosphaeria*, *Meyerozuma*, *Saccharomyces*, *Wickerhamomyces*	Kamilari et al. [79]
Greek-style	Kalamata	(former) *Lactobacillus*, *Pediococcus*, *Curvibacter*, *Sphinghomonas*, *Pichia*, *Brettanomyces*, *Issatchenkia*, *Cladosporium*	Michailidou et al. [81]
Natural green	Aloreña de Málaga	*Celerinatantimonas*, *Pseudomonas*, *Modestobacter*, *Propionibacterium*	Medina et al. [71]
Natural green	Nocellara Etnea	*Halomonas*, *Achromobacter*, *Marinobacter*, *Serratia*, *Bradyrhizobium*, (former) *Lactobacillus*	Randazzo et al. [73]
Natural green	Aloreña de Málaga	(former) *Lactobacillus*, *Pediococcus*, *Marinilactibacillus*, *Celerinatantimonas*, *Salinicola*, *Marinobacter*, *Pseudomonas*, *Vibrio*	Rodriguez-Gomez et al. [72]
Natural green	Aloreña de Málaga	*Zygotorulaspora*, *Pichia*, *Penicillium*, *Candida*, *Saccharomyces*, *Debaryomyces*, *Cladosporium*	Arroyo-Lopez et al. [70]
Spanish-style and natural green	Manzanilla	*Lactiplantibacillus*, *Alkalibacterium*, *Enterococcus*, *Serratia*, *Allidiomarina*, *Halomonas*, *Marinobacter*, *Pseudomonas*, *Saccharomyces*, *Pichia*, *Nakazawaea*, *Candida*	Ruiz-Barba et al. [74]
Natural green	Gordal, Manzanilla and Hojiblanca	*Lactiplantibacillus*, *Pediococcus*, *Halomonas*, *Marinobacter*, *Alidiomarina*, *Klebsiella*, *Kosakonia*, *Pseudomonas*, *Candida*, *Wickerhamomyces*, *Pichia*, *Nakazawaea*, *Saccharomyces*	Ruiz-Barba et al. [75]
California-style	Hojiblanca and Manzanilla	*Lactobacillus* (former), *Acetobacter*, *Vibrio*, *Oenococcus*, *Enterococcus*, *Streptococcus*, *Lactococcus*, *Alteromonas*, *Marinomonas*, *Acinetobacter*, *Shewanella*, *Kregervanrija*, *Pichia*, *Dispodascus*, *Magnusiomyces*, *Dekera*	Medina et al. [82]
Dry-salted	Throuba Thassos	*Candida*, *Pichia*, *Wickerhamomyces*, *Aureobasidium*, *Ogataea*, *Hortaea*	Gounari et al. [22]

## 5. Metabolomics

Metabolomics is a powerful tool for the identification and the quantification of low-molecular-mass metabolites (<1500 Da) produced by the metabolism of any organism [83]. By profiling the metabolites present in table olives, researchers can gain insights into the factors that affect the quality, safety, and nutritional value of the product [84]. Metabolomics can also be used to study the impact of chemical and enzymatic conversions during fermentation on the volatile profile of table olives and to identify specific molecules that affect their aroma and flavor [38]. All the relevant metabolomics studies are summarized in Table 2.

Gas chromatography–mass spectrometry (GC–MS) has been used for many years to investigate the metabolic profile of table olives [38]. One of the first studies by Montaño et al. [85] identified cyclohexanecarboxylic acid as the compound responsible for the zapateria spoilage odor, a frequent type of spoilage in Spanish-style table olives, which seems to be caused by bacteria belonging to the genera *Clostridium* and *Propionibacterium*. GC–MS analysis can also be used to distinguish the volatile organic compound (VOC) profiles from different table olive processing types and olive varieties. Sabatini and Marsilio [86], using GC–MS analysis, showed that the VOC profile of Nocellara del Belice table olives changed significantly with the different processing styles applied (Spanish, Greek and Castelvetrano). Bleve et al. [76] investigated the metabolic and volatile profiles of Greek-style Conservolea and Kalamata olives using headspace solid-phase microextraction followed by GC–MS (HS-SPME–GC–MS). It was highlighted that the two olive cultivars notably differed in the concentration of organic acids, higher alcohols and fatty acids. Analyzing the results via principal component analysis (PCA), it was also noted that aldehydes were found to be closely associated with the initial stage of fermentation (30 days), while higher alcohols, such as isoamylalcohols and styrene, were linked to the middle stage of fermentation (90 days), which was characterized by yeasts. Finally, the last stage of fermentation (180 days), which was dominated by bacteria, was related to the presence of acetate esters and acetic acid. The volatile profile of Spanish-style olives was explored via HS-SPME–GC–MS by Cortés Delgado et al. [87], and the most abundant compounds was found to be p-creosol, phenylethyl alcohol, acetic acid, ethanol, benzyl alcohol, ethyl acetate, and (Z)-3-hexen-1-ol. Moreover, it was possible to differentiate the VOC profiles between the Gordal, Manzanilla, and Hojiblanca cultivars, but not between the locations where they were cultivated. These findings suggest that the growing conditions of the olive fruit had a negligible impact on the volatile composition of Spanish-style green table olives in comparison with the olive cultivar that was used. In contrast, Mikrou et al. [88] discriminated between different table olives cultivars (Kalamata, Conservolea, Halkidiki) using their volatile profile and the growing location using HS-SPME–GC–MS. The variation among the table olives of the three cultivars primarily occurred due to the elevated levels of trans-β-ocimene and ethanol in the cv. Kalamata, α-muurolene and α-farnesene in the cv. Conservolea, and guaiacol and 4-methylguaiacol in the cv. Halkidiki samples. Nanou et al. [89] identified 88 volatile compounds from Spanish-style Conservolea and Halkidiki table olives via SPME–GC–MS, reporting only quantitative and no qualitative differences between the VOC profiles of the two varieties. Benitez-Cabello et al. [90] reported that different starter cultures can have a distinctive effect on the volatile profile of Spanish-style Manzanilla table olives. In more detail, it was observed that the ethanol and acetic acid ester concentrations were higher in brines inoculated with yeasts than with LAB. On the other hand, brines inoculated with the *Lp. plantarum* Lpl15 strain exhibited significant production of 4-ethylphenol, a compound associated with the formation of unpleasant odors in fermented foods. Sansone-Land et al. [91] focused on California-style olives from the United States, Spain, Egypt, and Morocco, analyzing their volatile profile via GC–MS. The main constituents of all the samples were found to be nonanal, (E)-dec-2-enal, 3-methylbutanal, ethyl benzoate, octanal, 2-methoxyphenol, 2-methylbutanal and 2-methoxy-4-methylphenol, while β-damascenone was identified as the biggest contributor to the aroma profiles of almost all the samples. In a similar study with GC–MS, Lopez-Lopez et al. [92] examined the alterations in the volatile substances throughout the processing and storage of California-style Manzanilla and Hojiblanca olives. The preservation stage was characterized by the presence of ethyl acetate, methyl acetate, and ethanol, while 2-methylbutanal, 3-methylbutanal, 3-ethylpyridine, and 3-ethyl-4-methylpyridine were formed during the darkening process in all the samples. In the final product after sterilization, benzaldehyde, dimethyl sulfide, and ethyl acetate prevailed against the other volatile compounds in both cultivars. Nevertheless, there were observed variations in the volatile composition and content between the two end products, especially during the darkening and sterilization steps. In general, the Manzanilla cultivar exhibited higher stability for specific volatile compounds. Selli et al. [93] conducted an olfactometric analysis of black dry-salted table olives via gas chromatography–mass spectrometry–olfactometry (GC–MS–O), revealing the presence of 17 key aroma compounds, including alcohols, esters, carboxylic acids, ketone, terpene and lactone. Pino et al. [94,95] evaluated the effect of different % NaCl concentrations, starter cultures and duration of fermentation on the VOC profiles of Nocellara Etnea natural green table olives. The results proved that different salt contents slightly affected the VOC profiles of the olives, and the changes mostly depended on the time of fermentation and the inoculation with starter cultures. A multi-statistical approach was employed by Garrido-Fernandez et al. [96] to discriminate between Manzanilla Spanish-style table olives affected by butyric, sulfidic, and putrid spoilage. Results obtained using HS-SPME–GC–MS and different statistical techniques combined showed that butyric spoilage was strongly linked with methyl butanoate, ethyl butanoate, and butanoic acid, while sulfidic spoilage was associated with 2-propyl-1-pentanol and putrid spoilage with D-limonene and 2-pentanol. In another recent study, Ruiz-Barba et al. [74] analyzed the volatilome of Spanish-style and natural green Manzanilla table olives via HS-SPME–GC–MS and attempted to correlate the microbial communities with volatile compounds. Spearman’s correlation showed that *Lactiplantibacillus* in the Spanish-style table olives presented positive correlations, most importantly with acids and esters such as acetic acid, butanoic acid, nonanoic acid, benzoic acid, methyl lactate, methyl acetate, and ethyl acetate. Regarding natural olives, *Aliidiomarina*, *P. manshurica*, and *Nakazawaea* showed positive correlations with several acids, alcohols, esters, ketones and terpenes, most notably with carbitol, ethyl phenylacetate, α-terpineol, phenol, and 2,3-dihydrobenzofuran.

Over the past few years, several studies have been conducted for the analysis of the metabolic and volatile profiles of table olives using techniques based on liquid chromatography (LC). Melliou et al. [97] used ultrahigh-pressure liquid chromatography–MS/MS (UHPLC–MS/MS) to investigate phenolic and secoiridoid compounds in black ripe and dry-salted table olives, demonstrating that the olive variety and processing method have a strong influence on the profile of phenolic and secoiridoid compounds in the olives. Both cultivars (cv. Mission and cv. Throuba Thassos) of the dry-salted olives showed higher amounts of the compounds studied, while California-style processing led to a significant reduction in all the compounds. The phenolic profile of Greek-style fermented Bella di Cerignola olives was determined by D’Antuono et al. [98] using high-performance liquid chromatography with diode-array detection (HPLC–DAD) and LC–MS/MS techniques. HPLC analysis identified six phenolic compounds, namely hydroxytyrosol, tyrosol, verbascoside, isoverbascoside, luteolin, and apigenin. Then, LC–MS and LC–MS/MS analyses confirmed the presence of the above compounds and identified three more phenolic compounds (hydroxytyrosol acetate, caffeoyl-6′-secologanoside, and comselogoside). Selli et al. [93] analyzed the phenolic compounds in black dry-salted table olives using liquid chromatography coupled to diode-array detection and electrospray ionization tandem mass spectrometry (LC–DAD–ESI-MS/MS). The results revealed the presence of 20 major phenolic compounds, such as luteolin-7-glucoside, verbascoside, oleuropein and hydroxytyrosol. Kalogiouri et al. [99] successfully discriminated PDO Greek Kalamata table olives from similarly processed olives from Egypt and Chile via ultra-high-performance liquid chromatography–quadrupole time-of-flight tandem mass spectrometry (UHPLC-ESI–QTOF-MS/MS), identifying 26 responsible compounds that could be used as markers for this discrimination. A metabolomic approach through ultra-high-performance liquid chromatography–high resolution mass spectrometry (UHPLC/HR-MS) was used by Vaccalluzzo et al. [100] for the profiling of phenolic compounds during the fermentation of Nocellara Etnea table olives inoculated with two different *Lactiplantibacillus* strains (*Lp. plantarum* C11C8 and *Lp. plantarum* F3.5). UHPLC/HR-MS identified seven different phenolic compounds, while both strains managed to decrease the oleuropein concentrations and increase the hydroxytyrosol concentrations of the samples, although *Lp. plantarum* C11C8 demonstrated better capability in both cases.

Electronic noses (e-noses) are another valuable tool in the field of volatilomics in relation to table olives, as they are able to detect and identify the VOCs that are responsible for the characteristic flavor and aroma profile of table olives. An evaluation was conducted by Martin-Tornero et al. [101] of two olive cultivars using an e-nose to assess the presence of acrylamide, a carcinogenic contaminant [102] produced during the sterilization treatment of Californian-style black olives. Increasing the sterilization time resulted in a reduction in the phenolic compounds; however, it facilitated the synthesis of acrylamide. In addition, e-noses can also provide significant assistance in discriminating between spoiled table olives by their VOC profiles, and several relevant studies have been conducted. Sanchez et al. [103] used an e-nose in order to discriminate between fermentations with zapateria, butyric, putrid, and musty defects in Spanish-style table olives. The e-nose data were analyzed via PCA to group the samples according to their volatile profiles. A clear discrimination of the defects succeeded, even when the defects were combined in one sample. In another study, Sánchez et al. [104] attempted to distinguish Spanish-style table olives inoculated with spoilage molds from the control fermentations, and e-nose analysis proved to be sensitive enough to detect table olives inoculated with different strains of the same mold species. Abnormal fermentations pose a significant problem for the table olive industry due to degrading the quality of the product, such as zapateria, which results in an undesirable aroma [80,105]. Therefore, in such cases, it is important to find a way to cover this characteristic odor so that the product is not wasted. Sanchez et al. [106] tried to mask the zapateria defect in Spanish-style table olives via the addition of the commercial flavoring “Mojo picón” to make them suitable for consumption. E-nose analysis proved that only the addition of 8% “Mojo picón” flavor was able to mask the characteristic unpleasant odor of the zapateria defect.

**Table 2:** Metabolomics studies on table olives.

Processing Method	Technique	Variety	Compounds	Reference
N/A	GC and HPLC	N/A	Lactic acid, acetic acid, propionic acid, n-butyric acid, isovaleric acid, n-valeric acid, n-caproic acid, acetaldehyde, methanol, ethanol, 2-butanol, n-propanol, cyclohexanecarboxylic acid	Montaño et al. [85]
Greek-style	HPLC and HS-SPME–GC–MS	Cellina di Nardò and Leccino	Ethyl acetate, isoamyl acetate, ethyl hexanoate, ethyl octanoate, acetic acid, propanoic acid, 2-methylpropanoic acid, octane, toluene, styrene, trimethyl benzene, linalol, linalolox, cymene, α-pinene, 2-methyl propanal, 2-methyl butanal, hexanal, benzaldehyde, 1-propanol, 1-butanol, 2-methyl-1-propanol, hexanol	Bleve et al. [107]
Greek-style	HPLC and HS-SPME–GC–MS	Conservolea and Kalamata	Ethanol, glycerol, citric acid, lactic acid, acetic acid, propionic acid, phenolic compounds, aldehydes, ketones, alcohols, isoamylalcohols, terpenes, esters, styrene, guaiacol	Bleve et al. [76]
Spanish-style	HS-SPME–GC–MS	Gordal, Manzanilla and Hojiblanca	Phenylethyl alcohol, benzyl alcohol, (Z)-3-hexen-1-ol, ethanol, ethyl acetate, ethyl lactate, ethyl octanoate, ethyl hexanoate, acetic acid, propanoic acid, benzaldehyde, alcohols linalool, linalool oxide, α-terpineol, p-creosol, p-ethylguaiacol, phenol	Cortés Delgado et al. [87]
Greek-style and Spanish-style	HS-SPME–GC–MS	Kalamata, Conservolea, Halkidiki	Acetic acid, formic acid, propanoic acid, ethanol, 2-butanol, 1-propanol, 3-methyl-1-butanol 2-butanone, methyl acetate, ethyl acetate, methyl propanoate, propyl acetate, 3-methylbutyl acetate	Mikrou et al. [88]
Spanish-style	HS-SPME–GC–MS	Manzanilla and Hojiblanca	Propanoic acid, 1-propanol, isopropanol, 2-heptenal, propyl acetate, (E)- 2-decenal, methyl hexanoate, 1-heptanol, isobutanol, 1-butanol	Garrido-Fernandez et al. [108]
Spanish-style	HS-SPME–GC–MS	Manzanilla	Acetic acid, butanoic acid, methanol, ethanol, 1-butanol, isopentanol, 1-hexanol, (Z)-3-hexen-1-ol, benzyl alcohol, phenylethyl alcohol, methyl acetate, ethyl acetate, methyl butanoate, ethyl butanoate, p-creosol, phenol, 4-ethylphenol, dimethyl sulfide	Garrido-Fernandez et al. [96]
Spanish-style	GC–MS	Manzanilla	Methanol, 2-phenylethyl acetate, 3-methyl-1-butanol, 2-butanol, 1-butanol, isoxylaldehyde, 4-ethylphenol, methyl acetate, ethyl acetate, 1-hexanol, 2-phenylethanol, benzyl alcohol	Benitez-Cabello et al. [90]
Spanish-style	SPME–GC–MS	Manzanilla, Hojiblanca and Gordal	Phenylethyl alcohol, ethanol, 1-propanol, 2-butanol, benzyl alcohol, 1-heptanol, 1-octanol, p-creosol, 4-ethyl phenol, o-guaiacol, acetic acid, propanoic acid, isobutanoic acid, 2-methylbutanoic acid, butanoic acid, ethyl acetate, triacetin, propyl propanoate, propyl acetate, octane, decane, benzaldehyde, (E)-2-heptenal, linalool, α-terpineol, copaene, dimethyl sulfide	Sanchez et al. [109]
Spanish-style	SPME–GC–MS	Manzanilla, Gordal and Hojiblanca	Octanoic acid, nonanal, phenylacetaldehyde, ethanol (6), ethyl acetate, geraniol, benzyl alcohol, benzaldehyde, 1-propanol, propanoic acid, methyl propanoate, propyl propanoate, propyl acetate, methyl hydrocinnamate, heptanal (29), propyl benzoate, benzyl propanoate, ethyl hydrocinnamate, 1,4-dimethoxybenzene, pseudocumene, heptanoic acid	López-López et al. [110]
Spanish-style and natural green	HS-SPME–GC–MS	Manzanilla	Ethanol, (Z)-3-hexen-1-ol, isopentanol, isobutanol, phenylethyl alcohol, ethyl acetate, ethyl lactate, ethyl hexanoate, acetic acid, 2-methylbutanoic acid, hexanoic acid, octanoic acid, 2-pentanone, 2-heptanone, 2-nonanone, dimethyl sulfide, dimethyl sulfoxide, linalool, α-terpineol, β-damascenone	Ruiz-Barba et al. [74]
California-style	GC–MS	Manzanilla, Hojiblanca, Picholine	β-damascenone, 3-methylbutanal, 2-methylbutanal, 2-phenylethanol, ethyl benzoate, 2-methoxy-4-methylphenol, octanol	Sansone-Land et al. [91]
California-style	GC–MS	Manzanilla and Hojiblanca	Ethyl acetate, methyl acetate, ethanol, 2-methylbutanal, 3-methylbutanal, 3-ethylpyridine, 3-ethyl-4-methylpyridine, benzaldehyde, dimethyl sulfide, ethyl acetate	Lopez-Lopez et al. [92]
California-style and dry-salted	UHPLC–MS/MS	Manzanilla, Mission and Throuba Thassos	Oleuropein, oleuropein aglycone monoaldehyde, hydroxytyrosol, hydroxytyrosol-4-O-glucoside, oleoside methyl ester, 2,6-dimethoxy-p-benzoquinone, chlorogenic acid, rutin, verbascoside, luteolin-7-O-glucoside, o-coumaric acid	Melliou et al. [97]
Spanish-style	SPME–GC–MS	Conservolea and Halkidiki	Acetic acid, propanoic acid, propyl acetate, propyl propanoate, 2-butanol, p-methylguaiacol, ethyl propanoate, cymene, thymol, ethanol, 1-propanol	Nanou et al. [89]
Spanish-style and Greek-style	GC–MS	Nocellara del Belice	Ethyl-acetate, 2-butanone, ethanol, propyl-acetate, ethyl propanoate, 2-butanol, 1-propanol, isopentanol, acetic acid, propionic acid	Sabatini and Marsilio [86]
Natural green	SPME–GC–MS	Nocellara Etnea	Acetic acid, hexanoic acid, propionic acid, ethanol, isoamyl-alcohol, phenyl-ethyl alcohol, ethyl acetate, ethyl butanoate, ethyl propanoate, ethyl lactate, nonanal, benzaldehyde, octanal, creasol, guaiacol, phenol, 4-ethyl phenol	Randazzo et al. [73]
Natural green	GC–MS	Nocellara Etnea	Ethanol, isoamyl alcohol, phenyl-ethyl alcohol, ethyl acetate, ethyl lactate, butanoic-acid-2-methylester, nonanal, cresol, propionic acid, isobutyric acid	Pino et al. [94]
Natural green	GC–MS	Nocellara Etnea	Isoamylalcohol, phenylethylalcohol, ethyl-acetate, methyl 2-methylbutanoate, acetic acid, nonanal, benzaldehyde, creasol,	Pino et al. [95]
Natural green	HS-SPME–GC–MS	Gordal, Hojiblanca and Manzanilla	Ethanol, (Z)-3-hexen-1-ol, isopentanol, isobutanol, phenylethyl alcohol, ethyl acetate, ethyl lactate, methyl 2,5-dimethyl-3-furoate, ethyl hexanoate, acetic acid, 2-methylbutanoic acid, 3-methylbutanoic acid, hexanoic, octanoic acid, 2-pentanone, 2-heptanone, 2-nonanone, dimethyl sulfide, dimethyl sulfoxide, linalool, α-terpineol	Ruiz-Barba et al. [75]
Dry-salted	GC–MS–O and LC–DAD–ESI-MS/MS	Gemlik	Hydroxytyrosol, p-coumaric acid, tyrosol, caffeic acid, verbascoside, oleuropein, luteolin, ethyl propanoate, methyl 2-methylbutyrate, isoamyl alcohol, (Z)-3-hexenol, acetic acid, isobutanol, 2-methyl-butanoic acid, 3-hydroxybutanone	Selli et al. [93]
Greek-style	UHPLC–ESI-QTOF-MS/MS	Kalamata	Catechol, hydroxytyrosol, tyrosol, L-malic acid, quinic acid, flavonoids, fatty acids, oleuropein, verbascoside, isoacteoside, campneoside II,	Kalogiouri et al. [99]
Greek-style	HPLC–DAD and LC–MS/MS	Bella di Cerignola	Tyrosol, Verbascoside, hydroxytyrosol acetate, hydroxytyrosol, isoverbascoside, luteolin, apigenin, caffeoyl-6′-secologanoside, comselogoside	D’Antuono et al. [98]
N/A	UHPLC/HR-MS	Nocellara Etnea	Oleuropein, oleuropein aglycone, hydroxytyrosol, oleoside-methyl ester, a decarboxymethyl dialdehydic form of oleuropein aglycone, eleanolic acid, decarboxymethyl eleanolic acid	Vaccalluzzo et al. [100]
Californian-style	HPLC and e-nose	Hojiblanca and Manzanilla	Hydroxytyrosol, tyrosol, procyanidin B1, vanillic acid, oleuropein, verbascoside, p-coumaric, acrylamide	Martin-Tornero et al. [101]
Spanish-style	HS-SPME–GC–MS and e-nose	Carrasqueña	Acetic acid, propanoic acid, butanoic acid, pentanoic acid, hexanoic acid, isopropyl alcohol, benzyl alcohol, phenylethyl alcohol, phenol, creosol, octanal, propyl propionate	Sanchez et al. [103]
Spanish-style	GC–MS and e-nose	N/A	Acetic acid, propylene glycol, pentanoic acid, beta-pinene, 2,4-hexadienoic acid, p-cymene, gamma-terpinene, diallyl disulphide, 2,4-hexadienoic acid, cyclohexanocarboxylic acid, creosol, propyl 2,4-hexadienecarboxylate, cuminaldehyde, alpha-terpinen-7-al, allyl trisulfide	Sanchez et al. [106]
Spanish-style	SPME–GC–MS and e-nose	Carrasqueña	Acetic acid, propanoic acid, butanoic acid, 2-methyl-butanoic acid, 1-propanol, benzyl alcohol, farnesol, 3-methyl-butan-1-ol, 2-methoxy-phenol, phenylethyl alcohol, creosol, octanal, nonanal, n-propyl acetate	Sánchez et al. [104]

## 6. Proteomics

Proteomics is the large-scale analysis of the expression, structure, composition, function and interactions of all the proteins produced in a cell, tissue or organism, while on the other hand, metaproteomics is focused on the characterization of the complete protein content of a whole microbiome [111,112,113,114]. Understanding the complete set of proteins in an organism is crucial for gaining insights into biological processes and their underlying mechanisms [115]. Based on the studies available, proteomics analyses in the field of table olives remain relatively scarce and there is still plenty of room for further research. Proteomics studies on table olives have focused on the proteomic profiles of LAB isolated from table olives or brines. Pessione et al. [116] evaluated the extracellular proteomes of *Lp. plantarum* S11T3 and *Lp. pentosus* S3T60C isolated from fermented olives and their brines via two-dimensional electrophoresis (2-DE) and matrix-assisted laser desorption/ionization time-of-flight/time-of-flight (MALDI-TOF/TOF) mass spectrometry. Seven extracellular proteins were identified from *Lp. plantarum* S11T3E and another six from *Lp. pentosus* S3T60C. Most of the proteins identified are involved in adhesion mechanisms, indicating the potential ability of the strains to adhere to the gut mucosa. Casado Munoz Mdel et al. [117] used 2-DE and chip–LC–QTOF to analyze the proteomics responses of *Lp. pentosus* MP-10 isolated from the brines of Aloreña green table olives when exposed to different antibiotics and biocides. *Lp. pentosus* MP-10, when adapted to antibiotics and biocides via being exposed to sub-lethal concentrations, showed an over-expression of ribosomal proteins, glutamyl-tRNA synthetase, NADH peroxidase and a small heat-shock protein, suggesting a possible activation of survival mechanisms. Perez Montoro et al. [118] focused on the adhesion ability of 31 *Lp. pentosus* strains isolated from naturally fermented Aloreña green table olives using an immobilized mucin model. Three strains (CF1-43N, CF1-37N, CF2-20P) were selected for their adhesive capacity for proteomics analysis, and the results showed that the highly adhesive *Lp. pentosus* CF1-43N over-produced four moonlighting proteins involved in the glycolytic pathway, stress response and transcription, which were not or under-produced in the other strains. All the relevant proteomics studies are summarized in Table 3.

## 7. Transcriptomics

Transcriptomics is the study of the complete collection of transcripts produced by the genome of a single-species sample [119]. Analyzing the transcriptome of an organism is essential to fully understand the functions and regulation of its genome [120]. While transcriptomics studies have been on the rise in recent years, there is still significant potential for further research in the field of table olives. As of now, no transcriptomics studies have been conducted in the field of table olives, with the exception of two studies about the transcriptomic profiles of LAB strains pre-adapted in vegetable oils that were isolated from table olives. Alonso Garcia et al. [121] investigated the transcriptomic profile of pre-adapted in vegetable edible oils *Lp. pentosus* AP2-16 isolated from Aloreña green table olives, focusing on the molecular mechanisms involved in the adaptation. The comparative analysis between the olive-adapted and control strains, and between the olive oil-adapted and almond-adapted strains, revealed that 125 and 108 genes, respectively, were differentially expressed. The transcriptional changes that were detected indicate the altering of the strain’s metabolic pathways to maintain the energy balance, cell growth and functionality. In the second study, Alonso Garcia et al. [122] evaluated the transcriptional changes in *Lp. pentosus* CF2-10 pre-adapted to olive or sunflower oil when exposed to antibiotics. The adapted strain demonstrated an increased antibiotic minimum inhibitory concentration (MIC) and overexpression of the stress genes *rpsL*, *recA* and *uvrB*, indicating a possible rerouting of its metabolic pathways to efflux toxic molecules such as antibiotics.

## 8. Conclusions

Investigating and fully understanding the molecular mechanisms that control the process of fermentation have been among the top priorities of researchers in the field for several years. Culture-based techniques have several limitations, the most significant of which is their tendency to favor the isolation of some microorganisms over others. Moreover, these techniques fail to provide insights into the molecular mechanisms (e.g., gene expression, protein synthesis, and metabolic pathways) that may underpin the behavior of a fermenting microbiome. To overcome these challenges, omics and meta-omics approaches have started to be widely used in the field of food microbiology. They enable the comprehensive analysis of the genomic, transcriptomic, proteomic, and metabolic profiles of microorganisms, enhancing our understanding of their functionality and interactions. While single-omics approaches have limitations in capturing the overall dynamics, multi-omics approaches enable exploration of cellular processes and interactions in response to the different fermentation stages. Despite the available information about the application of omics and meta-omics technologies in the field of table olives, their practical use in table olive production remains largely unexploited and currently relies on analyzing the microbiome and its potential impact on the characteristics of the final product. Several defects can be attributed to specific microorganisms, such as the softening of the olive tissue due to the presence of pectinolytic yeasts, the formation of gas pockets by the presence of enterobacteria in high numbers or the development of the zapateria defect that can be promoted by high populations of *Propionibacterium* and *Clostridium* [20,80]. Thus, different omics approaches could find application in the early detection of such problems. In addition, omics and meta-omics applications have their own set of limitations and challenges in the field of food research. Traditional metagenomic sequencing cannot distinguish between DNA originating from living and dead organisms within a microbiome, a crucial aspect in the field of food microbiome research [123]. The existence of fats, proteins, and other compounds within food matrices can present difficulties in terms of the application of universal protocols for the application of omics and meta-omics. Furthermore, the microbial distribution in food is often heterogeneous, leading to potential sampling biases where a sample might not be representative of the whole food product. Metabolomics faces challenges concerning existing databases, primarily because only a limited portion of the overall metabolite pool has been identified and included in these databases, leaving several naturally occurring metabolites still undiscovered [124]. Beyond the currently established view of omics and meta-omics, they can be combined with additional molecular/in silico tools to yield even more information. An important example is the study by Perpetuini et al. [48], who used transposon mutagenesis to identify the genes of *Lp. pentosus* C11 that could be critical for growth in olive brines, as already described above.

In this review, we presented an in-depth analysis of metagenomic, metabolomic, proteomic, and transcriptomic studies concerning table olive research to date. To the best of our knowledge, there are no metaproteomic or metatranscriptomic studies on table olives, even though these approaches have already been used in other fermented foods such as kimchi, soybeans, and fermented fish [125,126,127,128,129]. Future multi-omics research on table olives can shed additional light on the connection between the microbial community composition and functionality [38,130,131]. The application of multi-omics techniques may remain difficult to explore due to their requirement for advanced bioinformatics tools and computational power [123]. Nevertheless, applying multi-omics approaches is the next chapter in the study of table olives, since they can reveal the factors affecting sensory traits and product safety and may ultimately lead to the identification of specific microorganisms or metabolites as biomarkers associated with these characteristics.

## Figures and Tables

**Table 3:** Proteomics studies on table olives.

Technique	Proteins	Reference
2-DE and MALDI-TOF/TOF	Muramidase, gamma-D-glutamate-meso-diaminopimelate muropeptidase, glyceraldheyde 3-phosphate dehydrogenase, transglycosylase, peptidase M23B	Pessione et al. [116]
2-DE and chip–LC–QTOF	Ribosomal proteins, glutamyl-tRNA synthetase, NADH peroxidase, small heat-shock protein	Casado Munoz Mdel et al. [117]
2-DE and LC–MS/MS	Glucosamine-6-phosphate deaminase, phosphoglycerate mutase, transcription elongation factor GreA, small heat-shock protein	Perez Montoro et al. [118]

## Data Availability

The data used to support the findings of this study can be made available by the corresponding author upon request.
